# Winemaking: Advanced Technology and Flavor Research

**DOI:** 10.3390/foods13121937

**Published:** 2024-06-19

**Authors:** Fernanda Cosme, Fernando M. Nunes, Luís Filipe-Ribeiro

**Affiliations:** 1 Biology and Environment Department, School of Life Sciences and Environment, University of Trás-os-Montes and Alto Douro, 5000-801 Vila Real, Portugal; 2 Chemistry Research Centre-Vila Real (CQ-VR), Food and Wine Chemistry Laboratory, University of Trás-os-Montes and Alto Douro, 5000-801 Vila Real, Portugal; fmota@utad.pt; 3 Chemistry Department, School of Life Sciences and Environment, University of Trás-os-Montes and Alto Douro, 5000-801 Vila Real, Portugal

Beginning in ancient times, human societies around the world continue to produce fermented beverages from locally available sugar sources [1]. This widespread practice is due to ethanol’s analgesic, disinfectant, and mind-altering properties, which satisfy thirst, facilitate relaxation, promote social cohesion, and enhance eating pleasure [2]. Furthermore, fermentation aids in preserving and enriching the nutritional value of foods and beverages [3]. Because of their recognized pharmacological, nutritional, and sensory benefits, fermented beverages have played a crucial role in the evolution of human culture and technology, driving advancements in agriculture, horticulture, and food-processing techniques [1,4].

Wine, one of the oldest known fermented drinks, is intricately linked to the culture and traditions of its region. Within the agribusiness economy, the wine sector stands as one of the most significant market segments [5]. In recent years, the winemaking process has adapted to meet consumer demands, which increasingly prioritize climate change, sustainability, and health concerns. As a result, several trends have emerged in the wine sector to address these demands. These include the production of low-alcohol or zero-alcohol wines, made without additives or with natural additives and enriched with health-promoting components such as antioxidants. For instance, the goal of producing wines without the use of sulfur dioxide (SO_2_) has been pursued for some time. SO_2_ is the most used additive in winemaking. Due to its antimicrobial action, antioxidasic and antioxidant effect, SO_2_ can protect grape juice and wine from various undesirable reactions and is thus considered essential by many in winemaking. However, both SO_2_ and sulfites are known food allergens, capable of inducing breathing difficulties, sneezing, hives, migraines, and other issues. Increased levels of SO_2_ can also affect the wine’s sensory characteristics [6]. Alternative methods have been explored for replacing SO_2_ in winemaking, including the incorporation of bacteriocins, wine phenolics, chitosan, and lysozyme, as well as physical techniques such as ultrasound, ultraviolet radiation, pulsed electric field (PEF), and high hydrostatic pressure (HHP). However, none have demonstrated effectiveness comparable to SO_2_ [7]. Therefore, ongoing and necessary research on this topic is essential.

Low- and zero-alcohol wines have significantly expanded in recent years, addressing the preferences of consumers seeking to decrease or eliminate alcohol consumption. This trend aligns with the preferences of health-conscious individuals. In this context, the wine industry is increasingly focused on promoting wines with reduced ethanol content. Various strategies, including viticulture and fermentation techniques, have been developed; however, the use of selected yeasts may offer a suitable alternative with minimal impact on the wine aroma complexity and quality [8]. Utilizing commercial starters of *S. cerevisiae* strains enhances the safety, simplicity, and microbiological stability of wine production, thereby preventing economic losses. However, this approach may diminish the typicity. Conversely, incorporating non-*Saccharomyces* strains, either independently or in co-fermentation with *S. cerevisiae*, can enrich wines by imparting complex flavors, imbuing them with a unique regional character, increasing the presence of healthy compounds, enhancing the primary and secondary aromas, stabilizing the color, and reducing the ethanol content [9]. Another compelling option for reducing alcohol consumption is the consumption of fruit wines. Apart from providing a novel and diversified drinking experience with their unique and distinct flavors, these wines are also characterized by their moderate to low alcohol content [10,11]. Fruit wines are frequently favored by young and middle-aged consumers, particularly young women [12,13]. This aligns with the growing consumer demand for fruit wines, indicating significant development potential for the fruit wine industry [12]. Winemaking can enhance the value of fruits for consumption and address the issue of the imbalance in fruit production and marketing [11,14]. Many of the polyphenols and other bioactive compounds in the source materials are bound to insoluble plant compounds. The winemaking process releases many of these bioactive components into an aqueous ethanolic solution, thus increasing their biological availability for absorption during consumption [15]. The emergence of co-fermentation, where different fruits are fermented together, is gaining popularity as a form of wine innovation. This approach is appealing for its experimental nature and its ability to create unique flavor combinations. Therefore, utilizing ripe fruits or their juices for fruit wine production is considered an attractive method of utilizing surplus and over-ripened fruits [16,17]. The production of fruit wines or their distillates can enhance the sustainability of food production by reducing food waste along the supply chain [11].

On the other hand, spirits represent around 50% of global alcohol consumption. Compared to other alcoholic beverages such as wine or beer, spirits have received relatively less attention and study. The technology involved in producing spirits is notably more complex than that required for wine or beer production [18]. The production process of distilled spirits typically involves fermenting various agricultural products containing carbohydrates, followed by distillation of the fermented mixture, aging, and blending [19]. Distilled liquors are classified based on the type of starch or sugar source used in their production. These categories include grape-based spirits such as brandy and cognac, grain-based spirits such as whiskey, bourbon, scotch, and baiju, root-based spirits such as rum made from sugarcane or molasses, and fruit-based spirits including fruit brandies such as peach brandy or apricot brandy.

The distilled spirit industry has embraced innovation to improve the production efficiency and explore new flavors, while still honoring traditional methods. Technological advancements have enhanced the precision of the fermentation and distillation processes. Moreover, the utilization of creative ingredients and techniques, such as barrel aging with unconventional woods and the incorporation of exotic botanicals, has broadened the spectrum of flavors. An emerging trend involves the production of organic and nonstandard spirit beverages, featuring plant-derived aromatic components. Novel aging techniques, including accelerated aging using oak wood fragments and physical methods such as ultrasonic waves and gamma irradiation, have also surfaced. Given the high competitiveness and growing consumer awareness, producers are urged to prioritize the quality of both established and novel spirit beverages [20,21].

Regardless of whether they are new trendy products or traditional ones, the overall quality of fermented beverages is primarily determined by their aromatic and sensory profile. This profile is closely linked to their typicity, region, and, most importantly, their ability to influence consumer purchasing decisions [22]. In fermented beverages, the sensory complexity is mainly influenced by three aromatic categories developed during the production process. Varietal aromas, characterized by terpenes, thiols, carotenoids, and norisoprenoids, originate from the raw materials containing carbohydrates used in production. Fermentation aromas, consisting of higher alcohols, terpenoids, volatile fatty acids, esters, and phenols, are formed by yeasts during alcoholic fermentation [23,24]. Aging aromas develop during maturation and bottle aging, including volatile phenols, furanic compounds, and acetals [25,26,27,28,29].

In this context, this Special Issue is dedicated to advanced technologies applied in winemaking and flavor research, including distilled spirits. Following the peer review process, six original research articles were included in this Special Issue of *Foods*.

The study by Sánchez-García et al. (contribution 1) analyzed the relationship between green transformational leadership and green innovation in the wine industry, with a focus on the mediating roles of green motivation and creativity. Using data from 196 Spanish wine companies, the results revealed a positive and significant relationship between green transformational leadership and green innovation. Furthermore, green motivation and green creativity were found to mediate this relationship. The study concluded that managers in the wine industry should foster employee motivation and creativity, particularly in ecological terms, to promote the development of environmentally friendly innovations.

Minimizing food loss extends beyond its effects on food security and hunger; it also imposes a significant burden on the environment. Addressing food loss provides a chance to mitigate these adverse environmental impacts and advance towards a more sustainable future. *A. arguta* is a climacteric respiration fruit with thin skin, making it hard to store. Therefore, processing it into fruit wine utilizes non-commercial fruits and overproduction to alleviate spoilage problems and increase the added value of the product. The purpose of the study by Wen et al. (contribution 2) was to provide a theoretical basis for scientifically understanding the flavor and chemical nature of the aroma characteristics of different varieties of *A. arguta* to improve the quality of its fruit wine and select winemaking fruit varieties. Modern research has concluded that raw materials are the main determinants of wine and fruit wine quality. The selection of varieties for winemaking should not only consider the nutritional value of fruit wines but also comprehensively consider the color, flavor, and other indicators. In this study, the basic physicochemical indicators, color, organic acids, volatile components, and sensory quality of original wines made from 10 *Actinidia arguta* varieties were examined and analyzed. The results showed significant differences in the wines’ quality from different varieties. Of these, ‘Kuilv’ had the highest vitamin C content, total phenol content, and best color; ‘Jialv’ had the highest total flavonoid content, followed by ‘Fenglv’; ‘Tianxinbao’ had the highest dry extract content; and ‘Fenglv’ had the highest volatile flavor content. The sensory evaluation showed that ‘Tianxinbao’ had the highest total aroma score, followed by ‘Fenglv’. The comprehensive analysis revealed that ‘Kuilv’, ‘Fenglv’, and ‘Tianxinbao’ were more suitable for winemaking. In line with this trend, the study by Bezerra et al. (contribution 3) explored the production of high-value red fruit spirits from low-commercial-value red raspberries, blueberries, and strawberries. The fermentation process achieved high ethanol conversion yields without needing nutrient supplementation or fruit juice solid separation. The distillation produced fruit spirits that, except for the blueberry spirit with a lower volatile compound concentration, met the legal requirements for ethanol and methanol concentrations and had favorable aromatic profiles. This study demonstrates that fermenting and distilling these red fruits are efficient, consistent, and reproducible processes, creating high-quality spirits suitable for various market applications, including low-alcohol options, base spirits, or high-alcohol spirits through re-distillation.

*Lachancea thermotolerans* stands out among non-*Saccharomyces* and *Saccharomyces* yeasts as the only yeast capable of effectively acidifying wine during alcoholic fermentation. In addition to its acidification ability, it offers other secondary advantages, such as reducing the volatile acidity, consuming malic acid, decreasing the final ethanol concentration, and increasing the concentration of desirable volatile compounds such as ethyl phenyl acetate. However, there are important limitations to consider, including its moderate fermentative power and limited resistance to SO_2_. The limited SO_2_ resistance of *L. thermotolerans* can be addressed by using alternative compounds instead of SO_2_. Some of these alternatives can inhibit spoilage microorganisms such as bacteria or *Brettanomyces/Dekkera* and prevent oxidation while allowing *L. thermotolerans* to carry out fermentation. Chitosan emerges as a promising option to combine with *L. thermotolerans* and other non-*Saccharomyces* yeasts unaffected by this antimicrobial agent. The study by Vicente et al. (contribution 4) investigated the influence of chitosan on wines fermented with various strains of *L. thermotolerans*. Chitosan did not exhibit a significant impact on the fermentative power of *L. thermotolerans*, but it did significantly affect several other kinetic and chemical parameters of enological relevance. Notably, chitosan demonstrated a significant influence in increasing various acidification-related parameters, including lactic acid production, total acidity, and a pH reduction for all the studied strains of *L. thermotolerans*. Therefore, chitosan represents an intriguing tool for enhancing the acidification potential of *L. thermotolerans*. Additionally, chitosan significantly influenced other enological parameters such as malic acid consumption, PAN, i-butanol, 3-methylbutanol, and lactic acid ethyl ester. The impact of chitosan exhibited considerable strain variability, dependent on the specific *L. thermotolerans* strain under investigation. These findings highlight the multifaceted influence of chitosan on various enological parameters, emphasizing the importance of strain selection when employing chitosan in *L. thermotolerans* fermentations.

Baijiu is the national liquor of China, which has been produced for more than 2000 years. Abundant raw materials, multi-strain co-fermentation, and complex processes contribute to the mysteries surrounding baijiu flavor and taste, which still have not been fully explored. Wu et al. (contribution 5) reviewed the role of acid substances in baijiu. Acid substances significantly influence baijiu’s flavor and taste and possess functional qualities. Therefore, studying acids in baijiu as the core of the interaction between the flavor and taste of baijiu may be one of the important directions for future research. Notably, exploring the changes in microorganisms during baijiu fermentation is extremely complex. To better understand the relationship between microbial metabolites and flavor substances in baijiu fermentation, acid-producing microorganisms can be used as a starting point for exploration, which is of great significance to baijiu production practices. Finally, it is worth considering whether acid substances can be studied and confirmed as key factors of baijiu through practical application in the future. Generally, the aging process of baijiu is predominantly dark, with a storage time ranging from 2 to 5 years [25,26,27,28]. To shorten the aging time of baijiu, distilleries have innovated a new aging technique based on practical experience. The new aging technique involves exposing raw baijiu to the sun during the aging process. Practical experience has shown that sun-exposed baijiu exhibits improved softness. This improvement may result from transformations among trace components in baijiu (i.e., alcohols, aldehydes, acids, and esters); however, the mechanisms behind these changes are unknown. Further exploration is warranted to determine whether the concentration of long-chain fatty acid ethyl esters changes and how it influences baijiu flavor quality. The work of Wu et al. (contribution 6) on modern flavor sensomics and multivariate chemometrics has demonstrated that long-chain fatty acid ethyl esters play a significant role in the attributes of “softness”, “remaining taste”, and “aroma sensation in the mouth” of baijiu. Additionally, it has been shown that the longer the storage time of baijiu, the better the quality. Sun-exposure can accelerate the aging process of baijiu and reduce the storage costs, offering a new idea for baijiu production. By harnessing the effects of the sun, energy can be conserved and the production costs reduced.

The research papers published in this Special Issue represent some of the innovative strategies available for innovation in the wine industry, including the optimization of the production process, alternative fermentation technologies, and the use of different raw materials to produce new products, while also enhancing sustainability in the wine and spirit industries.
